# Positive RT-PCR tests among discharged COVID-19 patients in Shenzhen, China

**DOI:** 10.1017/ice.2020.134

**Published:** 2020-04-16

**Authors:** Xiujuan Tang, Shi Zhao, Daihai He, Lin Yang, Maggie H. Wang, Yuan Li, Shujiang Mei, Xuan Zou

**Affiliations:** 1 Shenzhen Center for Disease Control and Prevention, Shenzhen, China; 2 JC School of Public Health and Primary Care, Chinese University of Hong Kong, Hong Kong, China; 3 Department of Applied Mathematics, Hong Kong Polytechnic University, Hong Kong, China; 4 School of Nursing, Hong Kong Polytechnic University, Hong Kong, China

*To the Editor—*According to the current guideline of the National Health Commission of China, discharge of inpatients with the coronavirus 2019 (COVID-19) infection in China have to fulfill 2 recovery criteria: (1) symptoms disappear and computed tomography (CT) images become normal and (2) test negative for 2 consecutive times in reverse transcriptase-polymerase chain reaction (RT-PCR) tests for SARS-CoV-2.^1^ However, Lan et al^1^ recently reported 4 cases who were tested positive for SARS-CoV-2 at 5 days after discharge, suggesting positive status among discharged patients.^2^ To date, the prevalence and associated risk factors remain unclear.

We investigated all 209 patients with laboratory-confirmed SARS-CoV-2 infection who were discharged from the designated hospital in Shenzhen, China, between January 23 and February 21, 2020. Demographic data, laboratory profile, clinical data, and CT images were collected from these patients’ electronic medical records. Throat swabs and anal swabs were collected from all patients for RT-PCR tests according to the following scenarios: (1) on February 18, 2020, for those discharged before February 12, 2019; (2) on February 19, 2020 for those discharged between February 13 and 19, 2019; (3) on days 7 and 14 after discharge thereafter. This study was approved by the Shenzhen Center for Disease Control and Prevention review board and the need for informed consent was waived. All data used in this work are available upon request and approval of Shenzhen Center for Disease Control and Prevention.

We compared the settings in the study by Lan et al^2^ with those in this study (Appendix Table S1 online). Logistic regression models were adopted to explore the factors associated with the RT-PCR test results. Odds ratios (ORs) were calculated for the probability of positive test in throat swabs, or anal swabs, or either, and the rest were considered negative in each of the 3 scenarios. The results are as follows:

- Scenario 1: 9 positive RT-PCR test results from throat swabs
- Scenario 2: 13 positive RT-PCR test results from anal swabs
- Scenario 3: 22 positive RT-PCR for test results from either throat or anal swabs

Normally, only scenario 3 should be considered, but we included scenario 1 to be consistent with Lan et al.^2^

Among all 209 discharged patients, 9 (4.3%) tested positive in throat swabs only, 13 patients (6.2%) tested positive in anal swabs only, and 22 (10.5%) tested positive in either. Together, 10.5% of discharged patients showed virus shredding around an average of 4.7 days after discharge (range, 2–13 days). Under scenario 3, the logistic regression models revealed that a high risk of positive test was significantly associated with older age (OR, 0.95; 95% confidence interval [CI], 0.93−0.98), diarrhea during hospital stage (OR, 10.44; 95% CI, 1.60−68.16). The “during disease” stage was the other significant factor, with an adjusted and 9.59 (95% CI, 2.02−45.62) under scenarios 2 and 3, respectively. Expectoration during the disease stage is also a significant factor, with an adjusted OR of 4.00 (95% CI, 1.24−12.88) but only under scenario 3 (Table 1).

**Table 1.** Summary of the Characteristics of Study Patients and the Estimated Association Between the Individual Features and RT-PCR Testing Outcomes

		Throat Swabs			
Scenario 1		Positive (*N* = 9)	Negative (*N* = 200)	Crude OR	Adjusted OR^a^
Sex	Male	3	88	0.64 (0.15–2.71)	0.99 (0.27–3.62)
Age, y	Median (IQR)	32 (28–36)	45 (32–57)	0.97 (0.93–1.00)	0.95 (0.92–0.99)
Sampling delay, d	Median (IQR)	2 (2–2)	6 (3–7)	0.41 (0.21–0.81)	0.36 (0.18–0.72)
Symptoms	Dry cough	4	72	1.42 (0.36–5.65)	1.38 (0.44–4.32)
	Expectoration	2	27	1.83 (0.35–9.67)	3.03 (0.61–15.14)
	Cough	5	89	1.56 (0.39–6.19)	1.56 (0.52–4.70)
	Diarrhea	1	9	2.65 (0.28–24.89)	7.01 (0.52–95.40)

		Anal Swabs			
Scenario 2		Positive (*N* = 13)	Negative (*N* = 196)	Crude OR	Adjusted OR^a^
Sex	Male	5	86	0.80 (0.25–2.61)	0.83 (0.28–2.46)
Age, y	Median (IQR)	25 (6–39)	45 (32–58)	0.95 (0.91–0.98)	0.95 (0.92–0.98)
Sampling delay, d	Median (IQR)	7 (5–7)	5 (2–7)	1.04 (0.91–1.19)	1.11 (0.97–1.26)
Symptoms	Dry cough	6	70	1.54 (0.48–4.91)	1.92 (0.65–5.63)
	Expectoration	3	26	1.96 (0.49–7.87)	3.00 (0.67–13.37)
	Cough	8	86	2.05 (0.63–6.67)	3.12 (0.97–10.07)
	Diarrhea	2	8	4.27 (0.78–23.55)	10.44 (1.60–68.16)

		Either Throat or Anal Swabs			
Scenario 3		Positive (N=22)	Negative (N=187)	Crude OR	Adjusted OR^a^
Sex	Male	8	83	0.72 (0.28–1.83)	0.70 (0.29–1.66)
Age, y	Median (IQR)	28 (20–38)	46 (32–59)	0.95 (0.93–0.98)	0.95 (0.93–0.98)
Sampling delay, d	Median (IQR)	4 (2–7)	5 (3–7)	0.90 (0.77–1.05)	0.95 (0.81–1.10)
Symptoms	Dry cough	10	66	1.53 (0.61–3.81)	1.89 (0.81–4.39)
	Expectoration	5	24	2.00 (0.66–6.08)	4.00 (1.24–12.88)
	Cough	13	81	1.89 (0.75–4.75)	2.70 (1.12–6.51)
	Diarrhea	3	7	4.06 (0.93–17.64)	9.59 (2.02–45.62)

Note. RT-PCR, reverse transcriptase-polymerase chain reaction; OR, odds ratio; IQR, interquartile range.

a The OR is adjusted by the age, sex, sampling delay, disease severity and the backgrounds of the healthcare staff who delivered the treatment.

Although the prevalence of virus was substantial (10.5%), no infection was discovered among close contacts. Discharged COVID-19 patients in Shenzhen are required to be self-isolated for an additional 14 days after discharge to prevent the possible transmission due to the positive test post discharge.

Although live SARS-CoV-2 virus has been found in stool samples in some cases,^3^ the role of fecal–oral transmission remains unclear. Among 209 patients, 10 (4.8%) had diarrhea, and this ratio is slightly higher than the 3.8% rate based on 1,099 patients nationwide,^4^ and 2 of 10 patients (20%) with diarrhea showed positive tests post discharge with positive anal swabs. We report that 15.7% of patients <50 years old showed positive tests, while 2.4% of patients >50 years old showed positive tests from anal swabs. The delay between discharge and RT-PCR result date was negatively associated among positive cases of throat swabs, with an adjusted OR of 0.36 (95% CI, 0.18−0.72). This finding implies that the risk of positive tests gradually vanishes over time.

Our study was limited by the lack of treatment information. Further and large-scale study on this phenomenon is warranted. Nevertheless, this study sheds lights on the viral dynamics of COVID-19.

## Supplementary material

For supplementary material accompanying this paper visit http://dx.doi.org/10.1017/ice.2020.134. click here to view supplementary material
