# Supplementary material for: Positive RT-PCR tests among discharged COVID-19 patients in Shenzhen, China
Source: Infect Control Hosp Epidemiol. 2020 Apr 16:1–2. doi: 10.1017/ice.2020.134 (PMC7205550; doi:10.1017/ice.2020.134)
Supplement: Supplementary file 1 [file S0899823X20001348sup001.docx]

## **Appendix Table 1.** Comparison of the Settings Used by Lan et al ^2^ and in the Present Study

| Comparison | Lan et al^2^ | This Study |
| --- | --- | --- |
| No. of baseline cases | Not clear | 209 |
| No. of relapse cases | 4 | 22 |
| Occupation | Medical personnel | General population |
| Gender, M:F | 2:2 | 8:14 |
| Age range, y | 30−36 | Median, 28 (IQR, 20−38); range, 2−55 |
| Isolation (hospital:home) | 1:3 | 17 : 5 |
| Location | Wuhan, Hubei, China | Shenzhen, Guangzhou, China |
| Discharge criteria | Same as in CNHC^1^ | Same as in CNHC^1^ |
| Test delay post discharge, d | 5 | Median, 3.5 (IQR, 2−7); range, 2−13 |
| Swab method | Throat swabs | Throat swabs and anal swabs |
| Repeated test | Yes (≥3 tests per person) | Yes (≤2 tests per person) |

Note. IQR, interquartile range. CNHC, China National Health Commission.
